# Hospitalization costs of pediatric acute appendicitis in Western Romania: a single-center retrospective study

**DOI:** 10.25122/jml-2026-0037

**Published:** 2026-03

**Authors:** Ciprian-Ioan Borca, Madalin-Marius Margan, Corneluta Fira-Mladinescu, Alexandru Cristian Cindrea, Roxana Margan, Cristiana-Smaranda Ivan, Alexandru Alexandru, Ovidiu Alexandru Mederle, Sergiu Bajan, Brigitha Vlaicu, Vlad Laurentiu David

**Affiliations:** 1 Doctoral School, Victor Babes University of Medicine and Pharmacy, Timisoara, Romania; 2 Department of Pediatric Surgery and Orthopedics, Louis Turcanu Emergency Children’s Hospital, Timisoara, Romania; 3 Discipline of Public Health, Department of Functional Sciences, Victor Babes University of Medicine and Pharmacy, Timisoara, Romania; 4 Center for Translational Research and Systems Medicine, Faculty of Medicine, Victor Babes University of Medicine and Pharmacy, Timisoara, Romania; 5 Discipline of Hygiene, Department of Microbiology, Victor Babes University of Medicine and Pharmacy, Timisoara, Romania; 6 Center for Studies in Preventive Medicine, Victor Babes University of Medicine and Pharmacy, Timisoara, Romania; 7 Emergency Department, Emergency Clinical Municipal Hospital, Timisoara, Romania; 8 Department of Surgery, Victor Babes University of Medicine and Pharmacy, Timisoara, Romania; 9 Discipline of Pediatric Surgery and Orthopedics, Department of Pediatrics, Victor Babes University of Medicine and Pharmacy, Timisoara, Romania

**Keywords:** acute appendicitis, hospitalization costs, healthcare resource utilization, length of stay, postoperative complications, cost analysis, AA, acute appendicitis, CI, confidence interval, COVID-19, coronavirus disease 2019, CT, computed tomography, ED, emergency department, EUR, euro, GBD, Global Burden of Disease, ICD, International Classification of Diseases, LOS, length of stay, MRI, magnetic resonance imaging, OR, odds ratio, RON, Romanian leu, SBO, small bowel obstruction, US, ultrasound, ZAR, South African rand

## Abstract

Acute appendicitis is the most common pediatric surgical emergency, and a complicated disease associated with greater morbidity and resource use. However, institution-level cost data from Central and Eastern Europe remain limited. This study aimed to quantify the direct hospitalization costs of pediatric acute appendicitis in Western Romania and to assess whether disease severity, postoperative complications, and selected diagnostic or therapeutic factors were associated with higher inpatient costs. We conducted a single-center retrospective study including 1,535 pediatric admissions for acute appendicitis treated surgically between 1 January 2016 and 31 December 2025 at Louis Turcanu Children’s Hospital, Timisoara, Romania. Of these, 501 (32.6%) were classified as complicated appendicitis, and 971 (63.3%) patients were male. Median age was 11.08 years (IQR 8.58–14.50). Median length of stay was 6 days (IQR 4–8) overall, but was significantly longer in complicated than in non-complicated appendicitis (8 [7–11] vs. 5 [4–7] days). Median total hospitalization cost was 2,160 RON (IQR 1,406–3,356) overall, increasing from 1,710 RON (IQR 1,187–2,541) in non-complicated cases to 3,364 RON (IQR 2,432–4,618) in complicated cases. Cluster analysis identified four cost profiles; the highest-cost cluster had a median cost of 3,810 RON and the greatest proportion of complicated cases. Admissions with complications also had higher median costs than those without complications (3,441 vs. 1,806 RON) and longer hospitalization (8 vs. 5 days). Complicated pediatric acute appendicitis, therefore, imposes a substantially greater hospitalization burden, with disease severity and length of stay as the principal cost drivers.

## Introduction

Acute appendicitis remains the most common indication for urgent abdominal surgery in children and adolescents, accounting for a large proportion of pediatric emergency department (ED) presentations for acute abdominal pain and a substantial share of pediatric surgical admissions [1,2].

Clinical diagnosis is challenging, particularly early in the disease course, because symptoms may be non-specific and overlap with common pediatric conditions (gastroenteritis, mesenteric adenitis, urinary pathology, gynecologic causes in adolescents) [1,3]. Delayed diagnosis and treatment are consistently associated with increased likelihood of perforation and downstream infectious complications [1,4,5]. International practice guidelines emphasize that appendicitis severity at presentation (uncomplicated vs. complicated/perforated) is a major determinant of postoperative course and resource use, including need for prolonged antibiotics, additional imaging, and interventions for intra-abdominal infection [5].

Contemporary Global Burden of Disease (GBD) analyses confirm that appendicitis remains common worldwide, with marked geographic heterogeneity in both incidence and outcomes [6]. The incidence of appendicitis in children and adolescents was estimated at 109 per 100,000 in 2021, representing approximately 2.19 million new cases and comprising 12.93% of all appendicitis diagnoses across age groups [7]. Acute appendicitis is rare under the age of 5, accounting for about 5% of cases, but the risk of perforation escalates inversely with age [8]. Disease severity at presentation critically influences outcomes and resource use in children. Perforation rates in the U.S. have risen from 317.5 to 457.7 per 1,000 pediatric appendicitis cases between 2001 and 2015, resulting in approximately 25,000 annual perforations [4]. Globally, perforation occurs in 20-74% of pediatric cases, often exceeding 30%, and is more frequent in younger children (up to 61.3% in the 1-5 age group) due to diagnostic delays [9]. Complicated appendicitis, including perforation or abscess, disproportionately affects minorities, low-income groups, and those with government insurance, with rates as high as 48.6% in some cohorts [10]. These cases extend hospitalization (8.9 vs. 4 days for uncomplicated), increase revisit rates (22.9% vs. 8.9%), and elevate costs ($32,282 vs. $13,296) [11]. Delayed diagnosis further amplifies burdens, adding a 23% increase in adjusted costs (mean marginal cost of $2,712) and higher complication risks.

Direct hospital costs rise substantially in complicated/perforated disease compared with uncomplicated appendicitis, and costs increase further when care involves additional procedures, longer length of stay (LOS), and unplanned returns to care [12-15]. Postoperative intra-abdominal abscess—one of the most common complications after perforated appendicitis—is associated with measurable excess costs and resource utilization, including extended inpatient care and post-discharge encounters [14]. Intra-abdominal abscess post-appendectomy, occurring in 1-24% of cases (predominantly complicated), incurs adjusted costs of $27,394 vs. $15,586 for uncomplicated [14]. Beyond disease severity, studies across children’s hospitals demonstrate pronounced inter-hospital variation in diagnostic and treatment pathways and in the costs of appendicitis care, supporting appendicitis as a key target for value-based improvement efforts [16,17]. Implementation of standardized, evidence-based appendicitis care protocols has been associated with reductions in LOS and hospital costs, particularly for complicated appendicitis, without worsening patient-centered outcomes such as readmission or postoperative infection [18].

Diagnostic imaging is central to value-based care in suspected pediatric appendicitis because it affects diagnostic accuracy, timeliness, negative appendectomy rates, and downstream resource use [2,3]. Current guidance generally supports a staged pathway starting with ultrasound and reserving CT or MRI for equivocal cases, balancing high diagnostic performance against the radiation exposure of CT; evidence syntheses and prospective studies show that US, CT, and MRI can all achieve high accuracy when used within appropriate algorithms, with MRI performing comparably to CT with lower costs [19-23]. The CT–MRI choice is especially relevant in children because CT is fast and widely available but uses ionizing radiation, whereas MRI avoids radiation yet may introduce workflow and staffing constraints, making its economic impact highly context dependent; both comparative clinical studies (US±MRI vs CT pathways) and cost-effectiveness/costing analyses suggest that imaging strategy influences costs and throughput and should be evaluated within local resource conditions and practice patterns [24,25].

Unfortunately, in Romania, many future medical students have limited opportunities to participate in workshops that could boost motivation, knowledge, and practical experience that can improve their preparation for medical school.

While the cost and value of pediatric appendicitis care have been extensively studied in high-income health systems, there are comparatively fewer detailed, institution-level evaluations from Central and Eastern Europe that quantify hospitalization costs and identify local cost drivers under regional organizational constraints and reimbursement structures [26,27]. Moreover, “cost” is not interchangeable across systems: studies may report charges, reimbursements, or internally derived cost-accounting estimates, each reflecting different perspectives and limiting direct comparability between countries. Given known cross-institutional variation in appendicitis practice [16] and the evolving role of MRI-based pathways in pediatric emergency imaging [22,23], locally generated cost evidence is essential to guide resource allocation and protocol decisions. In low-resource settings like Eastern Europe, including Romania, perforation rates mirror global highs (18-48%), with limited data on costs. Romanian surveys indicate 92% of pediatric surgeons favor appendectomy for complicated cases, amid diagnostic challenges and variable imaging use [28]. During COVID-19, the proportion of complicated cases rose (e.g., a 4.4% increase globally [29]), reflecting delayed presentations and strained resources. In a Romanian case series, sporadic pediatric appendicitis amid SARS-CoV-2 infections highlighted management complexities, with higher complication risks [30].

The aim of this study was to quantify the direct hospitalization costs of pediatric acute appendicitis in Western Romania and to evaluate the extent to which disease severity, length of stay, and associated complications contribute to increased inpatient costs.

## Material and methods

**Figure 1:**
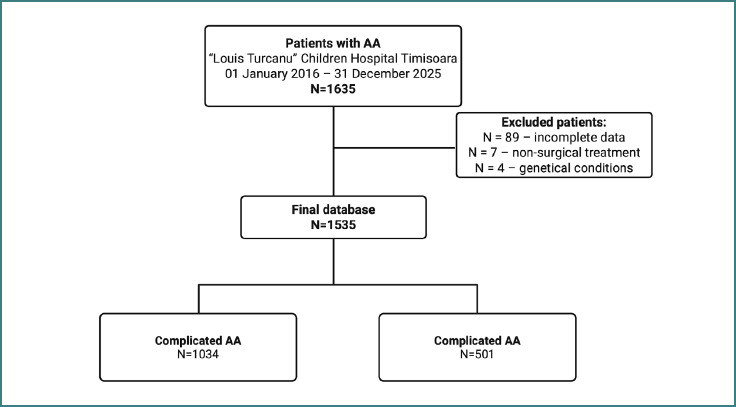
Study flowchart

A single-center, retrospective observational study was conducted at a regional tertiary care hospital in western Romania between 1 January 2016 and 31 December 2025. The initial study group consisted of all the patients diagnosed with acute appendicitis who were diagnosed and treated at the Louis Turcanu Children’s Hospital, Timisoara, between the given dates. Figure 1 shows the study flowchart.

The study protocol was reviewed and approved by the Ethics Committee of Victor Babes University of Medicine and Pharmacy, Timisoara, and the Ethics Committee of the Louis Turcanu Children’s Hospital, Timisoara.

The data were collected from the hospital’s electronic database, using ICD-10 diagnostic codes specific to acute appendicitis (K35) as both main and secondary diagnoses. To further extend the search, a keywords-based search was conducted. The identified patients were manually checked to ensure they met the inclusion and exclusion criteria.

Inclusion criteria were as follows: (1) age <18 years; (2) presence of acute appendicitis; (3) appendectomy during hospitalization time; (4) presence of at least one complication out of: post-appendectomy abscess, phlegmon, sepsis, hemorrhage, death; (5) no genetic immunocompromising conditions. The last criterion was used to avoid the confounding effect of the costs associated with the special needs of this patient population. Conversely, patients were excluded if any of the following were noted: (1) age above 18 years; (2) no surgical intervention; (3) pre-existing severe immunocompromised status; (4) incomplete data regarding the hospitalization costs or therapeutical approach.

The final database included patients’ sociodemographic characteristics, main and secondary diagnoses, and the case evolution. For each case, data from the patient’s expense account received at discharge were collected. The chart is divided into six categories: hospitalization expenses, food expenses, medication expenses, sanitary material expenses, laboratory expenses (including both labworks and imaging studies), and supplemental investigations.

Complicated appendicitis was defined for patients who presented with perforation, periappendiceal abscess, phlegmon, or generalized peritonitis.

Statistical analyses and visualizations were conducted in R (v4.5.1) using dplyr, tidyr, scales, stringr, patchwork, purrr, readxl, and ggplot2.

Prior to analysis, all data were de-identified and assessed for completeness and consistency. Flagged entries were verified against available metadata. Costs were analyzed in Romanian lei (RON) and reported in euros at a fixed conversion rate reflecting the 2025 average exchange rate (1 RON = 0.198 EUR); euro values are shown in square brackets throughout the manuscript.

Data normality was assessed using the Shapiro–Wilk test. The descriptive statistics encompass median and interquartile ranges for numeric variables, while categorical variables are described using counts and percentages. Between-group comparisons for continuous outcomes were performed using non-parametric tests (Mann–Whitney U/Wilcoxon rank-sum for two groups; Kruskal–Wallis for ≥3 groups with post-hoc pairwise Wilcoxon tests using Holm correction for multiple comparisons). Categorical variables were compared using Chi-square tests; when assumptions were not met, a Monte Carlo simulation (10,000 samples) was used.

To identify cost composition patterns, cost-component shares (component cost divided by total cost) were standardized and clustered using k-means (multiple random starts to improve stability). Cluster differences in costs and other clinical variables were evaluated using Kruskal–Wallis tests for continuous variables and Chi-square tests for categorical variables. Associations between selected postoperative/secondary diagnoses and total cost were examined using unadjusted comparisons relative to a baseline (“no complication” group) and multivariable linear regression on log-transformed total cost to account for right skewness.

Regression models included length of stay, age, sex, and admission year as covariates. Results are reported as β±standard error, *P* values, and as adjusted percent change in cost with 95% confidence intervals. Analysis of variance was conducted using partial F-tests.

Scenario analyses were performed to estimate potential savings associated with reductions in complicated appendicitis. The number of prevented cases was calculated under relative and absolute reduction scenarios, and savings were estimated by multiplying prevented cases by the observed incremental mean cost per complicated admission. Break-even intervention costs were calculated by dividing projected savings by the total number of appendicitis admissions.

All reported values are two-tailed, with *P* < 0.05 considered statistically significant. Where applicable, *P* values were adjusted for multiple comparisons using the Holm method.

## Results

### Descriptive statistics

Descriptive statistics are presented in Table 1. In total, 1,535 admissions for acute appendicitis were included, of which 32.6% were classified as complicated acute appendicitis. Overall, 971 patients (63.3%) were male. Median age was 11.08 years (IQR 8.58–14.50), with a slightly higher median age in the non-complicated group (11.42 [8.85–14.67]) compared with the complicated group (10.50 [7.42–14.17]). Median LOS for the entire cohort was 6 days (IQR 4–8) and was longer among complicated cases (8 [7–11]) than non-complicated cases (5 [4–7]). Complicated appendicitis had substantially higher median total costs (3,364 vs. 1,710; median incremental cost: 1,654), corresponding to a 1.97× increase.

**Table 1:** Descriptive statistics

Year	All patients (n = 1535)	Non-complicated AA (n = 1034)	Complicated AA (n = 501)
Male sex	971 (63.3%)	650 (62.9%)	321 (64.1%)
Age	11.08 (8.58-14.5)	11.42 (8.85-14.67)	10.5 (7.42-14.17)
LOS	6 (4-8)	5 (4-7)	8 (7-11)
Total cost	2,160 (1,406-3,356) [428 (2378-664)]	1,710 (1,187-2,541) [339 (235-503)]	3,364 (2,432-4,618) [665 (481-914)]

Abbreviations: AA, acute appendicitis; LOS, length of stay.

**Figure 2:**
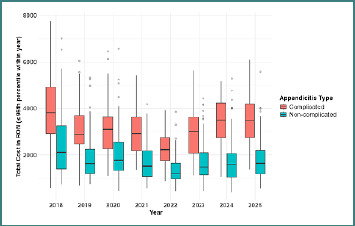
Yearly distribution of hospitalization costs for complicated and non-complicated appendicitis (outliers removed)

Year-stratified analyses (Table 2) demonstrated persistently higher costs in complicated appendicitis across the study period. Median costs for non-complicated cases ranged from 1,194 to 2,113 RON (approximately 234–414 EUR), while complicated cases ranged from 2,233 to 3,819 RON (approximately 438–749 EUR) depending on the year. Figure 2 illustrates the yearly distribution of hospitalization costs after removal of the upper 5% outliers to improve visualization of the central cost patterns. Across all years, complicated appendicitis consistently had higher median costs and greater cost dispersion than non-complicated cases.

**Table 2:** Yearly cost evolution of non-complicated AA vs complicated AA

Year	Non-complicated AA	Complicated AA	Median difference
2018	2,113 (1,396–3,266) [414 (274–640)]	3,819 (2,929–4,926) [749 (574–966)]	1,706 [335]
2019	1,626 (1,205–2,248) [319 (236–441)]	2,889 (2,472–3,698) [566 (485–725)]	1,263 [248]
2020	1,775 (1,332–2,555) [348 (261–501)]	3,100 (2,270–3,646) [608 (445–715)]	1,325 [260]
2021	1,520 (1,065–2,194) [298 (209–430)]	2,924 (2,197–3,638) [573 (431–713)]	1,404 [275]
2022	1,194 (966–1,650) [234 (189–324)]	2,233 (1,758–2,745) [438 (345–538)]	1,039 [204]
2023	1,485 (1,142–2,099) [291 (224–412)]	3,000 (2,084–3,632) [588 (409–712)]	1,515 [297]
2024	1,597 (1,039–2,064) [313 (204–405)]	3,505 (2,741–4,244) [687 (537–832)]	1,908 [374]
2025	1,644 (1,187–2,207) [322 (233–433)]	3,497 (2,745–4,197) [686 (538–823)]	1,853 [363]

* All the differences were statistically significant, with *P* values <0.001, the Mann–Whitney U statistical test. Abbreviations: AA, acute appendicitis.

Complicated appendicitis was associated with higher costs across all major hospital resource categories. The largest contributor to the incremental cost difference was hospital stay, accounting for 35.1% of the additional expenditure (median 1,657.57 vs 979.60). Medication costs were the second-largest contributor, accounting for 25.6% of the incremental cost (889.85 vs 394.42), followed by laboratory investigations (20.6%; 835.99 vs 437.17) and surgical materials (17.0%; 618.43 vs 289.84). Food services and imaging contributed minimally to the overall cost difference, accounting for 2.2% and 0.8% of the incremental cost, respectively.

### Cluster analysis

**Figure 3:**
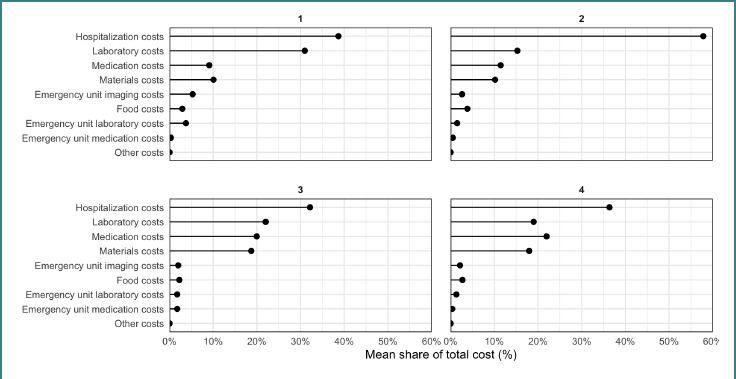
Lollipop graphs for the mean share of total cost for each of the 4 clusters

Cost composition clustering of 1,535 admissions identified four profiles (Figure 3). Cluster 1 (*n* = 311) and Cluster 2 (*n* = 685) were predominantly non-complicated (83.7% and 73.9%, respectively) and had the lowest median total costs: 1,390.43 RON [275.31 EUR] (IQR 1,024.07–2,195.34 RON [202.76–434.68 EUR]) for Cluster 1 and 1,876.33 RON [371.51 EUR] (IQR 1,358.85–2,776.67 RON [269.05–549.78 EUR]) for Cluster 2.

Higher-cost profiles included Cluster 4 (*n* = 667; 46.2% complicated) with a median cost of 3,140.62 RON [621.84 EUR] (IQR 2,117.53–4,223.10 RON [419.27–836.17 EUR]) and Cluster 3 (*n* = 46; 65.0% complicated) with the highest median cost of 3,810.44 RON [754.47 EUR] (IQR 3,311.64–5,338.49 RON [655.70–1,057.02 EUR ]).

Two low-cost profiles were predominantly non-complicated: Cluster 1 (*n* = 311; 83.7% non-complicated) and Cluster 2 (*n* = 685; 73.9% non-complicated). Cluster 1 showed a higher relative laboratory cost share (hospital 0.387; labs 0.310) and had the lowest median total cost (1,390.43 RON [275.31 EUR]; IQR 1,024.07–2,195.34 RON [202.76–434.68 EUR]) with the shortest LOS (4 days; IQR 3–6). Cluster 2 was dominated by hospitalization costs (hospital share 0.578) and had a higher median cost (1,876.33 RON [371.51 EUR]; IQR 1,358.85–2,776.67 RON [269.05–549.78 EUR]) and longer LOS (6 days; IQR 5–8). Two higher-cost profiles were enriched for complicated appendicitis. Cluster 4 (*n* = 667; 46.2% complicated) demonstrated a mixed composition (hospital 0.364; meds 0.220; labs 0.190; materials 0.180) with a median total cost of 3,140.62 RON [621.84 EUR] (IQR 2,117.53–4,223.10 RON [419.27–836.17 EUR]) and LOS 7 days (IQR 5–9). Cluster 3 (*n* = 46; 65.0% complicated) represented the highest-intensity profile with elevated shares across multiple components (hospital 0.322; labs 0.220; meds 0.200; materials 0.187), the highest median total cost (3,810.44 RON [754.47 EUR]; IQR 3,311.64–5,338.49 RON [655.70–1,057.02 EUR]) and LOS 8 days (IQR 6.25–10), and a younger median age (8.08 years; IQR 5.4–13.5).

Total hospitalization costs differed significantly across clusters (*P* < 0.001). Pairwise comparisons indicated that total cost differed significantly between all clusters (all adjusted *P* < 0.001). Cluster membership was significantly associated with appendicitis severity (Pearson’s chi-square coefficient = 114.92, *P* < 0.001).

### Most frequent secondary diagnostics impact on costs

**Figure 4:**
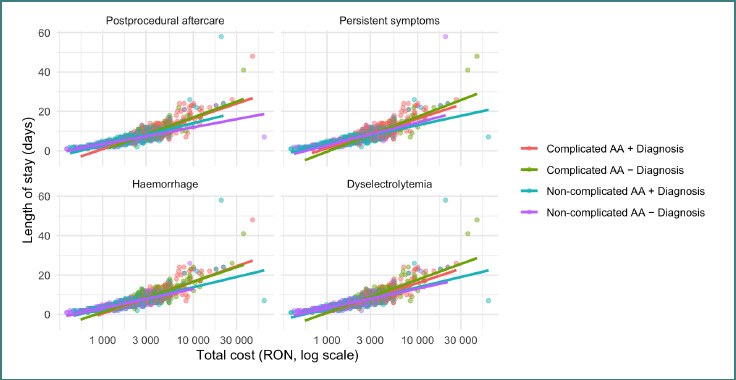
Scatter plots for the total cost and length of stay stratified by the most frequent diagnosis

Total cost was primarily associated with appendicitis severity and length of stay. In multivariable linear regression on log-transformed cost, LOS (*P* < 0.001) and complicated appendicitis (*P* < 0.001) were the strongest predictors. Adding secondary diagnosis indicators (“post-procedural aftercare”, “persistent symptoms”, “Hemorrhage”, “Dyselectrolytemia”) improved model fit statistically (partial F-test *P* < 0.001) but with a small increase in explained variance (Nagelkerke R^2^ 0.736 to 0.746). In the adjusted model, “Hemorrhage” and “Dyselectrolytemia” were independently associated with higher costs (+9.5% and +8.8%, respectively), “post-procedural aftercare” showed a smaller association (+5.3%), while “persistent symptoms” was not significant. Stratified analyses showed “Dyselectrolytemia” remained significant among complicated cases (~+8.8%), whereas R58 and “Dyselectrolytemia” were significant among non-complicated cases. In the adjusted log-cost model, secondary diagnoses were associated with modest but significant increases in total cost: post-procedural aftercare (+5.3%, 95% CI +1.3% to +9.4%), “Hemorrhage” (+9.5%, 95% CI +5.7% to +13.4%), and “Dyselectrolytemia” (+8.8%, 95% CI +5.2% to +12.6%), while “persistent symptoms” was not significant (+2.3%, 95% CI, −1.5% to +6.2%). These findings are graphically represented in Figure 4.

### Complication diagnostics impact on costs

Complications listed as secondary diagnoses were selected based on frequency and medical relevance. All these patients were part of the “complicated AA” subgroup. Among 1,535 admissions, 71.7% had none of the selected postoperative complication diagnoses, while 434 (28.3%) had at least one selected complication. Admissions with any complication had higher costs and longer LOS than baseline (median cost: 3,440.58 RON [681.23 EUR] vs 1,805.63 RON [357.51 EUR]; LOS: 8 vs 5 days; Wilcoxon *P* < 0.001). When categorized exclusively, total costs differed across groups (*P* < 0.001), with the highest costs observed in multiple-complication admissions (median 3,892.49 RON [770.71 EUR]; LOS 9 days), followed by sepsis-only and ileus/obstruction-only admissions. These findings are summarized in Table 3.

**Table 3:** Complications associated with the median length of stay and costs

Variable	n (%)	Median LOS	Median costs
No complication	1101 (71.7%)	5 (4-7)	1,805 (1,241-2,719) [357 (246-538)]
Persistent fever	108 (7%)	6 (5-9)	2,241 (1,437-3,354) [444 (285-664)]
Severe anemia	36 (2.3%)	7 (6-9)	3,066 (2,295-3,963) [607 (454-784)]
Ileus/obstruction	78 (5.1%)	8 (6-11)	3,304 (2,365-4,897) [654 (468-969)]
Sepsis	68 (4.4%)	8 (7-9)	3,805 (3,138-4,559) [754 (621-902)]
Multiple complications	144 (9.4%)	9 (7-12)	3,892 (3,183-5,958) [771 (630-1,180)]

**Figure 5:**
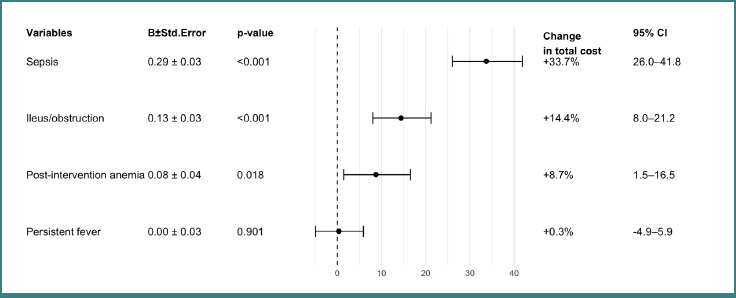
Forest plot for the impact of complications on costs

In multivariable log-cost regression adjusted for LOS, age, sex, and year, LOS was the dominant predictor of cost (*P* < 0.001). Among the postoperative complication diagnoses, sepsis (*P* < 0.001), ileus/obstruction (*P* < 0.001), and post-intervention anemia (*P* = 0.018) remained independently associated with higher costs, while persistent fever was not (*P* = 0.901). These findings are detailed in Figure 5.

### Scenario-based savings

Based on the observed mean hospitalization costs and case distribution, the total hospital expenditure for appendicitis admissions during the study period was estimated at approximately 4.26 million RON [843,000 EUR]. Using a mean incremental cost of 1,932 RON [382 EUR] per complicated admission, the estimated excess cost burden of complicated appendicitis was 967,932 RON [191,651 EUR] (22.7% of the total spending) over the study period. Scenario analyses suggested that a 5% and 10% relative reduction in complicated cases would yield gross savings of 48,396 RON [9,582 EUR] and 96,793 RON [19,165 EUR], respectively, while absolute reductions of 5 and 10 percentage points would generate savings of 148,281 RON [29,360 EUR] and 296,562 RON [58,719 EUR] (Table 4). The break-even intervention cost ranged from 32–193 RON per patient [6.3–38.2 EUR], depending on the magnitude of reduction achieved.

**Table 4:** Estimated excess cost burden and scenario-based savings. Mean incremental costs per complicated admission were 1,932, with an estimated excess cost burden of 967,932.

Scenario	Prevented cases	Savings	Break even
Relative 5%	25.05	48,396 RON [9,582 EUR]	32 RON [6.3 EUR]
Relative 10%	50.10	96,793 RON [19,165 EUR]	63 RON [12.5 EUR]
Absolute 5pp	76.75	148,281 RON [29,360 EUR]	97 RON [19.2 EUR]
Absolute 10pp	153.50	296,562 RON [58,719 EUR]	193 RON [38.2 EUR]

At the population level, this burden corresponds to an average additional cost of approximately 631 RON [125 EUR] per appendicitis admission, or roughly 63,057 RON [12,486 EUR] in excess costs per 100 admissions attributable to complications.

## Discussion

In this 10-year single-center retrospective study from Western Romania, one in three pediatric appendicitis admissions was complicated (32.6%), boys predominated (63.3%), and complicated disease almost doubled direct inpatient costs compared with non-complicated appendicitis (median 3,364 vs. 1,710 RON), while prolonging LOS from 5 to 8 days. Cost clustering and multivariable modeling converged on the same conclusion: severity at presentation and bed-day use were the principal determinants of expenditure, whereas secondary diagnoses and postoperative complications added smaller but still measurable cost increments.

The male sex was more frequent in this cohort (63.3% of the patients), similar to other international cohorts [11]. The overall median age of 11.1 years aligns with the usual school-age-to-early-adolescent peak described in global pediatric appendicitis epidemiology [1,6,7]. The proportion of complicated appendicitis was 32.6%, higher than the 24.9% rate reported in a Central European survey by Dotlacil *et al*. [26] but still falls within the 20–74% range reported in literature [9].

The younger age of children with complicated disease in our cohort (10.5 vs. 11.4 years) also mirrors prior work showing an inverse relation between age and perforation risk [8,9]. Pogorelić *et al*. [8] reported that among children aged 5 years or younger, 64.4% already had perforation, with hospitalization of 7.5 versus 5 days in perforated versus non-perforated cases, respectively. In a prospective pediatric study, complicated appendicitis was also more frequent in children younger than 5 years (10.5% vs. 1.4%), further supporting age as a marker of late presentation and advanced disease [31]. Consequently, a meaningful part of the excess cost of complicated appendicitis could be caused by a delayed or less specific presentation in younger children, rather than with operative management alone. A direct significant association could not be found in the current cohort.

The consistent step-up in LOS and costs with increasing disease severity is notable. In our cohort, complicated appendicitis increased median LOS by 60% (8 vs. 5 days) and median cost by 96.7% (3,364 vs. 1,710 RON). In the multicenter study by Anandalwar *et al*., the most severe intraoperative findings were associated with a 2.2-fold difference in LOS (4.0 vs. 8.9 days) and a 2.4-fold difference in mean cumulative cost ($13,296 vs. $32,282) [11]. Ferguson *et al*. [14] similarly showed that postoperative intra-abdominal abscess after pediatric perforated appendicitis added 6.1 bed days and raised adjusted mean hospital costs from $15,586 to $27,394. In a resource-limited setting, Kong *et al*. [32] reported a gradient from 6,578 ZAR in uncomplicated appendicitis to 14,791 ZAR with localized sepsis and 34,773 ZAR with generalized sepsis. Although absolute values are not directly comparable because our study used Romanian direct hospitalization costs rather than US charges or broader 30-day cumulative costs, the relative cost gradient is strikingly consistent across settings.

Our findings demonstrate that complicated appendicitis is associated with substantially higher hospitalization costs compared with non-complicated disease, with an estimated incremental cost of approximately 1,654 per admission in our cohort. The median cost of complicated over non-complicated appendicitis persisted every year and ranged from 1,039 to 1,908 RON, corresponding to roughly 1.75- to 2.19-fold higher annual median costs. This temporal stability suggests that severity is a more durable determinant of cost than year-specific drift in prices or practice.

The year-stratified analyses in our series extend that point: the median cost premium for complicated over non-complicated appendicitis persisted across years and ranged from 1,039 to 1,908 RON, corresponding to roughly 1.75- to 2.19-fold higher annual median costs. This temporal stability suggests that severity is a more durable determinant of cost than year-specific drift in prices or practice.

Our cluster analysis showed a simple gradient: children in the highest-intensity group were younger, had complicated appendicitis more often, stayed longer in the hospital, and generated the highest costs, while those in the lowest-intensity group had the shortest stays and the lowest costs. This pattern is consistent with previous studies showing that greater clinical severity is associated with greater resource use. For example, in complicated appendicitis, parenteral nutrition increased adjusted 30-day costs by 22.9% without improving outcomes [16]. Standardized evidence-based care pathways, on the other hand, reduced costs for complicated appendicitis by 24.6% and also shortened length of stay [18]. Even among children with non-perforated appendicitis, more severe intraoperative findings were associated with greater postoperative resource use, including more imaging (5.8% vs. 3.7%) and longer hospitalization (1.6 vs. 0.9 days) [33].

An additional insight from our clustering is that the lowest-cost profile had a comparatively larger laboratory share, whereas the higher-cost profiles were increasingly dominated by hospitalization, medications, and sanitary materials. These findings are consistent with the broader literature, which shows that the main financial penalty in appendicitis is usually prolonged inpatient care rather than additional diagnostic testing [14,19,20,34]. It is estimated that hospital room costs accounted for 66% of the excess cost after postoperative abscess [14]. Guideline-supported ultrasound-first selective-escalation pathways and contemporary imaging meta-analyses show that US, CT, and MRI can all achieve high accuracy when used appropriately, with cost-effectiveness analyses favoring risk-tailored staged imaging rather than screening advanced imaging for all patients [2,3,19].

After adjustment, LOS and complicated appendicitis remained the dominant determinants of total cost, whereas post-procedural aftercare (+5.3%), hemorrhage (+9.5%), and dyselectrolytemia (+8.8%) contributed smaller residual increments. The dyselectrolytemia finding is particularly plausible in light of the growing literature on sodium disturbances in appendicitis. A 2024 meta-analysis found that preoperative hyponatremia was associated with complicated appendicitis with a pooled OR of 4.11 [35]. Pediatric studies have shown hyponatremia in 73.3% to 89.5% of perforated cases, with serum sodium around 132.2 mmol/L in complicated versus 139.2 mmol/L in uncomplicated disease and AUC values near 0.98 for discrimination [31,36]. By contrast, appendicitis-specific pediatric cost data for postoperative hemorrhage and anemia are limited, but the broader surgical literature shows a similarly modest independent economic effect once baseline severity is accounted for: preoperative anemia increased hospital costs by 18% in pediatric spinal deformity surgery and by 14% in adult colorectal surgery [37,38].

In our study, any selected complication nearly doubled the median cost (3,440.6 vs. 1,805.6 RON) and increased the median LOS from 5 to 8 days; the most expensive categories were multiple complications and sepsis-only admissions. This mirrors the work of Ferguson *et al*., in which intra-abdominal abscess nearly doubled cost and increased readmission risk 7.8-fold [14]. Complicated appendectomy carried a 12.8% 30-day readmission rate and a median readmission-attributable cost of $6,524, representing a 53% relative increase in cumulative treatment cost [13]. Revisit rates range from 8.9% to 22.9%, and costs range from $13,296 to $32,282, according to intraoperative severity [11]. A recent systematic review found that complicated appendicitis approximately doubled the odds of later adhesive bowel obstruction (pooled OR 2.02), and long-term pediatric follow-up data showed that perforation and postoperative intra-abdominal abscess increased SBO risk 9.03-fold and 6.98-fold, respectively [39,40]. The lack of an independent adjusted effect for persistent fever in our model is likewise reasonable, as isolated fever may simply proxy inflammatory burden already captured by severity and LOS rather than act as a distinct economic driver.

Regional surveys show significant heterogeneity in perioperative antibiotics, drainage, laparoscopy, and complicated-case management across Central Europe, while the Romanian national survey found that 92.4% of pediatric surgeons perform appendectomy for complicated appendicitis, but minimally invasive uptake remains limited in this subgroup [26,28]. Our results indicate that, within such heterogeneous practice environments, the largest savings are likely to come from standardizing care, earlier recognition of severe disease, and reducing avoidable postoperative bed-days rather than from marginal reductions in single tests or materials. The relevance of these measures was underscored during the COVID-19 era, when the U.S. proportion of complicated pediatric appendicitis rose from 46.5% to 50.9%, and Romanian pediatric reports similarly highlighted management complexity during SARS-CoV-2 circulation [29,30].

Several interventions could potentially reduce the incidence of complicated appendicitis and thereby lower hospital costs. Clinical pathways aimed at early diagnosis, including standardized appendicitis scoring systems such as the Alvarado Score and the Pediatric Appendicitis Score, may improve early risk stratification and facilitate timely diagnostic evaluation in patients presenting with suspected appendicitis [41-43]. When integrated into structured emergency department pathways, these tools can support earlier clinical decision-making and reduce diagnostic delays. In addition, ultrasound-first diagnostic protocols have been shown to shorten time to diagnosis, decrease perforation rates, and minimize unnecessary imaging exposure [44,45]. Fast-track surgical pathways and early surgical consultation may further reduce delays to operative treatment, which is particularly important given evidence that delays in surgical management are associated with an increased risk of perforation and disease progression [46,47]. From an economic perspective, previous cost analyses have demonstrated that complicated appendicitis substantially increases healthcare resource utilization compared with uncomplicated disease; importantly, these findings have been validated in Chinese cohorts as well [48,49]. Because the break-even cost in our analysis ranged between 27 and 165 per patient, relatively low-cost interventions such as structured diagnostic protocols, clinical scoring systems, or ultrasound-based streamlined emergency department pathways could plausibly generate net cost savings while simultaneously improving patient outcomes [50–52].

This study should be interpreted in light of several limitations. As a retrospective analysis from a single tertiary pediatric center, the findings reflect local patterns, management strategies, and costing practices, which can limit direct extrapolation to other settings. In addition, the economic analysis was confined to direct in-hospital costs of the index admission, so the broader financial impact of appendicitis—such as outpatient care, later readmissions, or indirect family costs—was not assessed. As with any study based on routinely collected clinical and administrative data, some potentially relevant details were not consistently available for all patients. Finally, although the cluster analysis offered a useful way to explore patterns of clinical severity and cost, these profiles should be viewed as exploratory and would benefit from confirmation in future multicenter prospective studies.

## Conclusion

This single-center retrospective study demonstrates that complicated pediatric acute appendicitis imposes a substantially greater hospitalization burden than non-complicated disease in Western Romania, with markedly higher inpatient costs and longer length of stay. Complicated appendicitis was associated with nearly double the hospitalization cost and consistently higher expenditures across all study years. Disease severity and hospitalization duration emerged as the primary cost drivers, while laboratory testing, medications, and surgical materials contributed to additional cost increases. Cluster analysis further identified distinct cost profiles, with higher-cost clusters enriched for complicated appendicitis and characterized by longer hospital stays and more intensive resource utilization.

At the hospital level, complicated appendicitis accounted for approximately 22.7% of the total spending on appendicitis admissions, representing a substantial economic burden. Scenario analyses suggested that even modest reductions in the proportion of complicated cases could generate meaningful cost savings. If similar patterns were observed across other institutions, these findings may indicate a considerable economic burden at the health system level. These results highlight the potential clinical and economic benefits of strategies aimed at earlier diagnosis and timely surgical management in pediatric appendicitis.

## Data Availability

The data presented in this study are available upon request from the corresponding author.
